# Delayed Hemorrhage: A Rare Complication of Loop Electrosurgical Excision Procedure

**DOI:** 10.7759/cureus.53320

**Published:** 2024-01-31

**Authors:** Randy Felber, Hemangi Patel, Alyson Skelly, Alexandria Sobczak, Tanique Campbell

**Affiliations:** 1 Medical School, Nova Southeastern University Dr. Kiran C. Patel College of Osteopathic Medicine, Clearwater, USA; 2 Medical School, Nova Southeastern University Dr. Kiran C. Patel College of Osteopathic Medicine, Fort Lauderdale, USA; 3 Obstetrics and Gynecology, Wellington Regional Medical Center, Wellington, USA

**Keywords:** gynecologic surgical procedures, post-op complications, delayed hemorrhage, gynecology, oncological gynecology, leep, loop electrosurgical excision procedure

## Abstract

Loop electrosurgical excision procedure (LEEP) is a common procedure used to treat cervical dysplasia. It is performed by using an electrical current and loop wire to remove abnormal cervical tissue. Common complications include intraoperative and postoperative bleeding. A rare complication is delayed hemorrhage, presenting in 0.8-1.3% of women, which can require sutures, transfusions, or inpatient care. We present the case of a 41-year-old female presenting to the emergency department nine days after a LEEP procedure with heavy vaginal bleeding that resulted in delayed hemorrhage. Within hours of arrival, the patient passed several large clots and her hemoglobin dropped from 12.2 gm/mL to 6.9 gm/mL requiring emergency surgery and blood transfusion. The delayed hemorrhage was further exacerbated by this patient’s concurrent clopidogrel use. It is pivotal to identify high-risk patients to help prevent potential procedural complications through the use of preoperative instructions and emerging intraoperative interventions. It is also imperative to provide adequate guidance to patients about the postoperative course and how to identify signs and symptoms of a life-threatening situation.

## Introduction

Loop electrosurgical excision procedure (LEEP) involves removing the affected portion of the cervix and is the preferred treatment approach for high-grade cervical dysplasia. General complications of LEEP include intraoperative and postoperative vaginal bleeding [1-3]. A less common complication is delayed hemorrhage, or postoperative bleeding occurring at the procedure site several days to weeks after the operation [4]. Delayed hemorrhage after LEEP is not well documented in the literature and can require additional medical intervention and in rare cases, a trip to the emergency room.

To prevent perioperative or delayed hemorrhage, hemostasis tactics have been investigated. The use of local anesthesia including epinephrine and vasopressin has lowered the likelihood of perioperative or postoperative bleeding [4,5]. Thus, serious delayed hemorrhage requiring sutures, transfusions, or inpatient care is rare and only affects 0.8-1.3% of women who undergo LEEP [3,6,7]. We present the case of a 41-year-old female presenting with heavy vaginal bleeding after a LEEP procedure. We discuss the diagnosis and management of cervical laceration after a LEEP requiring multiple cervical sutures, surgical power, and blood transfusion.

## Case presentation

A 41-year-old female with a past medical history of coronary artery disease with stent placement, asthma, and cervical intraepithelial neoplasia (CIN) 2/3 presented to the emergency department (ED) via emergency medical services for heavy vaginal bleeding that started early that morning. Her recent surgical history is significant for a LEEP procedure. She restarted her Plavix (clopidogrel) and aspirin therapy on postoperative day one. She then noticed spotting of blood on postoperative day four. After noticing the spotting on postoperative day four, she discontinued her Plavix and aspirin therapy on her own. When she presented to the emergency department, she was on postoperative day nine from her LEEP procedure. On the day of presentation in the emergency room, she woke up with heavy vaginal bleeding that soaked through two pads per hour. In the emergency room, the patient admitted to shortness of breath but denied abdominal pain, sexual intercourse, nausea, vomiting, fever, chills, and chest pain. Her last menstrual period was 13 days ago, and her menses normally occur every 30 days and last three days. 

On the physical exam, her temperature was 98.4ºF (36.9ºC), heart rate of 118 beats per minute, respiratory rate of 20 breaths per minute, oxygen saturation was 100% on room air, and blood pressure of 134/97 mmHg. Routine labs showed a red blood cell count of 3.90 x10e6/mcL, hemoglobin of 12.2 gm/dL, hematocrit of 35.6%, and platelets of 237 x10e3/mcL. Urine analysis showed red-colored urine, moderate blood, red blood cells, and white blood cells. Further examination by the consulted obstetrics and gynecologist (OBGYN) showed normal appearing external genitalia, a large clot was expelled upon internal examination, as well as ~30cc of dark red blood was noted in the vaginal canal. Once the clot and blood were removed, a small site of active bleeding was noted at the six o'clock position of the cervix. This was immediately resolved with pressure, silver nitrate, and absorbable Surgicel hemostat powder. Hemostasis was achieved at the end of the exam and the patient was in stable condition. The nurse was instructed to notify the OBGYN if the patient saturated through one peri-pad before the planned reevaluation. 

Less than an hour later, the patient saturated through one peri-pad, and the OBGYN was notified. The patient exhibited mild distress and nervousness but did not display any signs of clinical deterioration related to blood loss. A repeat pelvic examination showed a large amount of dark red blood on the peri-pad with active cervical bleeding. Repeat hemoglobin was 10.9 gm/dL and hematocrit was 31.9%. Due to the continued cervical bleeding and the downtrend in hemoglobin, the OBGYN decided to proceed with an examination under anesthesia with potential surgical intervention.

The patient was taken directly to the surgical floor and preoperative labs were drawn showing a hemoglobin of 9.1 gm/dL and a hematocrit of 26.4%. While being prepped for surgery, the patient became pale, diaphoretic, and confused. She was rushed to the operating room where she was given general anesthesia. The anesthesiologist obtained another hemoglobin level in the operating room due to the patient’s rapidly declining state. This hemoglobin level was 6.9 gm/mL and verbally reported to the OBGYN over the phone as they were en route to the operating room. At that point, the patient had been closely monitored in the ED for eight hours. A unit of packed red blood cells was started and the patient was placed in the modified dorsal lithotomy position in Allen stirrups and prepped and draped in the normal sterile fashion. A speculum was placed in the patient's vagina and a large clot was noted in the vaginal canal; active cervical bleeding was noted at the six o'clock position. Multiple figure-of-eight stitches were placed using 3-0 chromic. Hemostasis was achieved. The cervical bed was then cauterized with the ball cautery and excellent hemostasis was obtained. Absorbable Surgicel hemostat powder was generously applied. After the speculum was removed, vaginal packing and a Foley catheter were inserted. A total of two units of packed red blood cells were given to the patient and an estimated 150 mL of blood was lost during the procedure.

**Figure 1:**
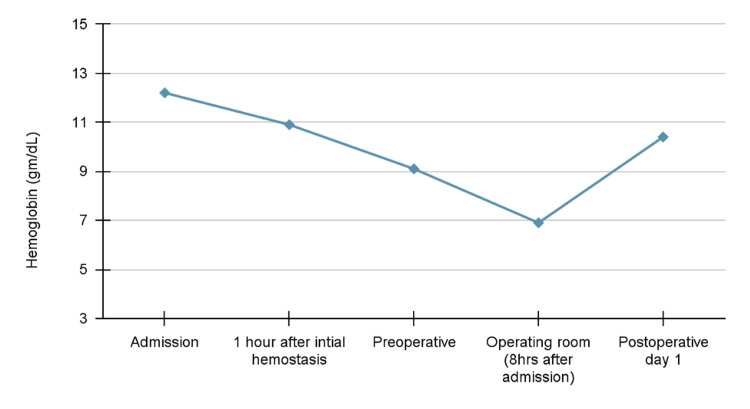
Hemoglobin over time - demonstrating the rapid decline in hemoglobin.

On postoperative day one, the patient was well appearing, ambulating, tolerating an oral diet, urinating, and without any overnight events. She also denied any nausea, vomiting, fevers, chills, chest pain, or shortness of breath. The Foley catheter and vaginal packing were removed and no bleeding was noted. Repeat labs showed a hemoglobin of 10.4 gm/mL and a hematocrit of 30%. The hemoglobin levels throughout the patient's hospital stay are illustrated in the graph below (Figure 1). The patient was then sent home and advised to follow up with her cardiologist and OBGYN.

## Discussion

The loop electrosurgical excision procedure is a common excisional procedure for the diagnosis and treatment of high-grade CIN. LEEP is completed using a wire loop using electric current to treat high-grade lesions of the cervix [2]. The advantage of a LEEP procedure compared to the cold knife or laser conization is the ease and time it takes to do the procedure and ultimately having a lower medical cost of the treatment of CIN [4].

A LEEP complication affecting only 0.8-1.3% of women is delayed hemorrhage, as seen in the presenting patient [3,6,7]. Depending on the percentage of blood lost, hemorrhage is categorized into four classes: class I is defined as up to 15% blood volume lost, class II as 15-30% blood volume lost, class III as 30-40% blood volume lost, and class IV as 40% blood volume lost or more. The most common symptoms include nausea, fatigue, pallor, cooling of extremities, tachycardia, tachypnea, delayed capillary refill, hypotension, and change in mental status. Severe hemorrhage can lead to shock which occurs when blood loss cannot meet the oxygen demands of the tissue leading to lactic acid production, organ failure, coma, and potentially death [8]. Thus, it is imperative to treat any hemorrhage symptoms immediately to avoid long-term consequences in patients.

Although the exact procedural steps used in our patient's LEEP are unknown, there are emerging intraoperative therapies that could help prevent postoperative hemorrhage. Epinephrine and vasopressin are powerful vasoconstrictors that can be combined with local anesthesia to reduce the incidence of major blood loss after a LEEP. An important consideration in patients undergoing a LEEP is the potential to inject vasopressin into the cervix before conization which can further decrease the incidence of delayed hemorrhage by controlling the blood loss. Routine use of these vasoconstrictors could help reduce the rates of delayed hemorrhage after a LEEP. In addition, hemostatic agents such as Beriplast, TachoComb, or TacoSil can be applied prophylactically to patients undergoing LEEP to reduce the likelihood of hemorrhage [4]. 

In this life-threatening case, the use of cervical sutures placed in a figure-of-eight was the only successful method to provide hemostasis. The rate of life-threatening hemorrhage requiring such interventions after a LEEP could be significantly reduced in the future with the use of emerging intraoperative therapies and careful postoperative management in high-risk patients.

## Conclusions

LEEP is a common procedure used to treat cervical dysplasia. This case reports severe delayed hemorrhage following a LEEP procedure, which is a serious and rare complication, affecting 0.8-1.3% of women. Calculating the risk of severe hemorrhage using possible risk factors should be a standard practice in gynecologic surgical procedures. This case highlights the importance of identifying preventable causes of postoperative complications, whether that’s reviewing pertinent medical history that may play a role in recovery, providing additional instructions on current antiplatelet therapy, or educating your patients on the early signs of delayed hemorrhage. Additionally, there should be a greater focus on being able to identify high-risk patients and when to utilize new intraoperative vasoconstrictive therapies to prevent delayed hemorrhage requiring blood transfusion.
